# Supplementary material for: Development and Validity of a General Nutrition Knowledge Questionnaire (GNKQ) for Chinese Adults
Source: Nutrients. 2021 Dec 3;13(12):4353. doi: 10.3390/nu13124353 (PMC8707636; doi:10.3390/nu13124353)
Supplement: Supplementary file 1 [file nutrients-13-04353-s001.zip › nutrients-1455382-supplementary.pdf]

Supplementary material: The C-GNKQ and its English translation.

Nutrition survey

This is a survey, not a test. Your answer will help identify which dietary advice people find confusing.

1. It is important that you complete it by yourself.
2. Your answers will remain anonymous.
3. If you do not know the answer, mark 'not sure' rather than guessing.

Section A. The first few items are about what advice you think experts are giving us.

1 Do you think health experts recommend that people should be eating more, the same amount or less of these foods?

	More	Same	Less	Not sure
Whole grain	☐	☐	☐	☐
Salty foods	☐	☐	☐	☐

2 Fat is one of the basic nutrients necessary for the human body.

☐ yes
☐ no
☐ not sure

3 The following substances are the main sources of energy that we need everyday.

	Yes	No	Not sure
Water	☐	☐	☐
Vitamins	☐	☐	☐
Mineral salt	☐	☐	☐

4 According to the experts, the calorie intake for breakfast, lunch, and dinner should be:

☐ 20%、50%、30%
☐ 30%、40%、30%
☐ 30%、30%、40%
☐ 20%、30%、50%

Section B. Experts classify foods into groups. We are interested to see whether people are aware of what foods are in these groups.

1 Do you think these are high or low in added sugar?

	High	Low	Not sure
Banana	☐	☐	☐
Yoghurt (sugary)	☐	☐	☐
Fruit juice (processed, concentrated)	☐	☐	☐

2 Do you think these are high or low in fat?

	High	Low	Not sure
Noodles (without sauce)	☐	☐	☐
Sesame sauce	☐	☐	☐
Salami	☐	☐	☐
Nuts	☐	☐	☐

3 Do you think these are high or low in protein?

	High	Low	Not sure
Squid	☐	☐	☐
Butter	☐	☐	☐

4 Do you think the foods listed below contain trans fatty acids?

	Yes	No	Not sure
Margarine	☐	☐	☐
Whole milk	☐	☐	☐
biscuits	☐	☐	☐
Fried sunflower seeds	☐	☐	☐
Olive oil	☐	☐	☐

Crisps	☐	☐	☐

5 Do you think these are high or low in fiber-roughage?

	High	Low	Not sure
Banana	☐	☐	☐
Eggs	☐	☐	☐
Beef	☐	☐	☐
Fish	☐	☐	☐
Chicken	☐	☐	☐

6 Do you think these fatty foods are high or low in saturated fat?

	High	Low	Not sure
Carp	☐	☐	☐
Minced meat	☐	☐	☐
Margarine	☐	☐	☐
Cream	☐	☐	☐

7 Do you think experts call these a healthy alternative to red meat?

	Yes	No	Not sure
Sausage	☐	☐	☐
Soybeans	☐	☐	☐
Nuts	☐	☐	☐
Low fat milk	☐	☐	☐
Eggs	☐	☐	☐

8 A glass of unsweetened fruit juice counts as a helping of fruit.

☐ Agree
☐ Disagree
☐ Not sure

9 There is more protein in a glass of whole milk than in a glass of skimmed milk.

☐ Agree
☐ Disagree
☐ Not sure

10 Which of these grains contain the most vitamins and minerals?

☐ Wheat
☐ Rice
☐ Millet
☐ Not sure

11 Harder fats contain more:

☐ Monounsaturated fatty acids
☐ Polyunsaturated fatty acids
☐ Saturates
☐ Not sure

12 Which of these foods contain the highest carbohydrate?

☐ Livestock and poultry meat
☐ Grains
☐ Vegetables and fruits
☐ Egg and milk
☐ Not sure

13 Which one of the following has the most calories for the same weight?

☐ Vitamins
☐ Protein
☐ Fat
☐ Carbohydrate
☐ Not sure

14 Do you think eating potatoes is one of the most economical and convenient ways to get calcium?
- ☐ Yes
- ☐ No
- ☐ Not sure

15 Do you think eating lean meat can promote iron absorption?
- ☐ Yes
- ☐ No
- ☐ Not sure

16 Comparing dark-colored vegetables and light-colored vegetables, which types of vegetables have a higher nutritional value?
- ☐ Dark-colored vegetables
- ☐ Light-colored vegetables
- ☐ Same
- ☐ Not sure

Section C. The next few items are about choosing foods. Please answer what is being asked and not whether you like or dislike the food.

1 Which would be the best choice for a low fat, high fiber light meal?
- ☐ Chicken legs
- ☐ Sweet potato, corn
- ☐ Mung bean congee
- ☐ Chicken pastry
- ☐ Not sure

2 Which of these would be the healthiest fried noodles with soy sauce?
- ☐ A large number of noodles with a little sauce on top
- ☐ A small number of noodles with a lot of sauce on top
- ☐ Not sure

3 If a person wanted to reduce the amount of salt in their diet, which would be the best choice?
- ☐ fried noodles with soy sauce
- ☐ Chicken soup with rice
- ☐ Chicken pastry
- ☐ Scrambled Eggs with Lettuce
- ☐ Not sure

Section D. This section is about health problems or diseases.

1 What diseases or health problems do you think are related to a low intake of fruit and vegetables?

	Yes	No	Not sure
Common cold	☐	☐	☐
Rheumatism	☐	☐	☐
Night blindness	☐	☐	☐

2 Do you think anemia is related to sugar?
- ☐ Yes
- ☐ No
- ☐ Not sure

3 Do you think night blindness is related to salt or sodium?
- ☐ Yes
- ☐ No
- ☐ Not sure

4 What diseases or health problems do you think are related to fat?

	Yes	No	Not sure
Ulcer	☐	☐	☐
Heart disease	☐	☐	☐

5 Do you think this help to reduce the chances of getting certain kinds of cancer?

	Yes	No	Not sure
Eating less fruit	☐	☐	☐
Eating less salt	☐	☐	☐
Eating less sugar	☐	☐	☐

6 Do you think these ways help prevent heart disease?

	Yes	No	Not sure
Eating more diet fiber	☐	☐	☐
Eating less salt	☐	☐	☐
Eating less saturated fat	☐	☐	☐
Eating less preservatives or additives	☐	☐	☐

7 Which one of these is more likely to raise people's blood cholesterol level?

☐ Fruits
☐ Animal fats
☐ Plant oils
☐ Grains
☐ Not sure

8 Have you heard of antioxidant vitamins?

☐ Yes
☐ No

9 If 'yes' to question 8, do you think these are antioxidant vitamins?

	Yes	No	Not sure
Vitamin C	☐	☐	☐
Vitamin D	☐	☐	☐
Vitamin E	☐	☐	☐
Vitamin K	☐	☐	☐

营养调查

这是一项调查，而不是测试。您的答案将有助于确定人们对哪些饮食建议感到困惑。

1.您必须独立完成。

2.您的答案将保持匿名。

3.如果您不知道答案，请选择“不确定”。

第一部分 前几个问题是关于你认为营养专家给我们的建议

1 你认为营养学专家建议人们食用以下食物的量是：

	多吃	适中	少吃	不确定
全谷物	☐	☐	☐	☐
高钠食物	☐	☐	☐	☐

2 脂肪是人体所必需的基本营养素之一吗？

☐ 是

☐ 不是

☐ 不确定

3 下列物质是我们每天所需能量的主要来源吗？

	是	不是	不确定
水	☐	☐	☐
维生素	☐	☐	☐
无机盐	☐	☐	☐

4 根据营养学专家的建议，一日三餐热量的合理供应要求早、中、晚餐的热能比例为？

☐ 20%、50%、30%

☐ 30%、40%、30%

☐ 30%、30%、40%

☐ 20%、30%、50%

第二部分 专家把食物分成不同种类，我们想了解人们是否知道各种类中有哪些食物。

1 以下食物的糖含量程度是：

	高	低	不确定
香蕉	☐	☐	☐
酸奶（含糖）	☐	☐	☐
橙汁（非鲜榨）	☐	☐	☐

2 以下食物的脂肪含量程度是：

	高	低	不确定
水煮面（无汤无酱）	☐	☐	☐
芝麻酱	☐	☐	☐
腊肠	☐	☐	☐
坚果	☐	☐	☐

3 以下食物的蛋白质含量程度是：

	高	低	不确定
鱿鱼	☐	☐	☐
黄油	☐	☐	☐

4 你认为下列食物含有反式脂肪酸吗?

	有	没有	不确定
人造黄油	☐	☐	☐
全脂牛奶	☐	☐	☐
饼干	☐	☐	☐
炒瓜子	☐	☐	☐
橄榄油	☐	☐	☐
薯片	☐	☐	☐

5 以下食物的膳食纤维含量程度是：

	高	低	不确定
香蕉	☐	☐	☐

鸡蛋	☐	☐	☐
牛肉	☐	☐	☐
鱼肉	☐	☐	☐
鸡肉	☐	☐	☐

6 你认为以下食物的饱和脂肪酸含量程度是：

	高	低	不确定
鲤鱼	☐	☐	☐
肉馅	☐	☐	☐
人造黄油	☐	☐	☐
奶油	☐	☐	☐

7 你认为以下食物是红肉的健康替代品吗？

	是	不是	不确定
腊肠	☐	☐	☐
大豆	☐	☐	☐
坚果	☐	☐	☐
低脂牛奶	☐	☐	☐
鸡蛋	☐	☐	☐

8 不加糖的果汁和水果的营养成分是一样的。
☐ 同意
☐ 不同意
☐ 不确定

9 一杯全脂牛奶的蛋白质含量比一杯脱脂牛奶的高。
☐ 同意
☐ 不同意
☐ 不确定

10 哪一种谷物中含有最多的**维生素和矿物质**?
☐ 小麦
☐ 大米
☐ 小米
☐ 不确定

11 较硬的脂肪含有更多:
☐ 单不饱和脂肪酸
☐ 多不饱和脂肪酸
☐ 饱和脂肪酸
☐ 不确定

12 下列哪类食物**碳水化合物**含量最高？
☐ 畜禽肉类
☐ 粮谷类
☐ 蔬菜水果类
☐ 蛋奶类
☐ 不确定

13 下列哪种营养素单位质量提供的**能量**最高？
☐ 维生素
☐ 蛋白质
☐ 脂肪
☐ 碳水化合物
☐ 不确定

14 吃土豆是最经济、最方便的**补钙方式**之一吗？
☐ 是
☐ 不是
☐ 不确定

15 吃瘦肉可以促进**铁**的吸收吗？

- ☐ 可以
- ☐ 不可以
- ☐ 不确定

16 深色蔬菜与浅色蔬菜比较，哪类蔬菜的营养价值较高?
- ☐ 深色蔬菜
- ☐ 浅色蔬菜
- ☐ 一样高
- ☐ 不确定

第三部分 这一部分是关于日常食物选择的，请根据题目而非您的饮食偏好作答。

1 以下哪一个食物是低脂高纤维的最好选择？
- ☐ 鸡腿
- ☐ 红薯、玉米
- ☐ 绿豆粥
- ☐ 鸡肉酥饼
- ☐ 不确定

2 以下哪一种炸酱面更健康？
- ☐ 大量的面条，上面只淋上一点炸酱酱汁
- ☐ 少量的面条，上面淋上很多炸酱酱汁
- ☐ 不确定

3 如果一个人想要减少饮食中盐的摄入量，那么以下哪一个是最好的选择？
- ☐ 炸酱面
- ☐ 鸡汤泡饭
- ☐ 鸡肉酥饼
- ☐ 莴笋炒鸡蛋

第四部分 这一部分是关于健康问题和疾病的。

1 你认为以下疾病或健康问题是否与**少吃水果和蔬菜**有关?

	有关	无关	不确定
感冒	☐	☐	☐
风湿	☐	☐	☐
夜盲症	☐	☐	☐

2 你认为贫血是否与**糖**有关?
- ☐ 有关
- ☐ 无关
- ☐ 不确定

3 你认为夜盲症是否与**盐或钠**有关?
- ☐ 有关
- ☐ 无关
- ☐ 不确定

4 你认为以下疾病或健康问题是否与**脂肪**有关?

	有关	无关	不确定
溃疡	☐	☐	☐
心脏病	☐	☐	☐

5 你认为以下做法是否能有助于**降低患某些癌症的几率**？

	是	不是	不确定
少吃水果	☐	☐	☐
少吃盐	☐	☐	☐
少吃糖	☐	☐	☐

6 你认为以下做法是否能有助于**预防心脏病**吗?

	是	不是	不确定
多吃纤维	☐	☐	☐
少吃盐	☐	☐	☐
少吃饱和脂肪	☐	☐	☐
少吃防腐剂或添加剂	☐	☐	☐

7 以下哪一种食物更有可能提高人们的**血胆固醇**水平?

- ☐ 水果
- ☐ 动物脂肪
- ☐ 植物油
- ☐ 谷物
- ☐ 不确定

8 你听说过抗氧化维生素吗？

- ☐ 听说过
- ☐ 没听说过

9 如果题目 8 的答案为听说过的话，你认为以下哪些是**抗氧化维生素**？

	是	不是	不确定
维生素 C	☐	☐	☐
维生素 D	☐	☐	☐
维生素 E	☐	☐	☐
维生素 K	☐	☐	☐

Scoring rules for the C-GNKQ

Section A:

1-1. More	1-2. Less	2. A	3-1. No	3-2. No	3-3. No	4. B

Section B:

1-1. High	1-2. High	1-3. High	2-1. Low	2-2. High	2-3. High	2-4. Low	3-1. High	3-2. Low	4-1. Yes
4-2. No	4-3. Yes	4-4. Yes	4-5. No	4-6. Yes	5-1. High	5-2. Low	5-3. Low	5-4. Low	5-5. Low
6-1. Low	6-2. High	6-3. High	6-4. High	7-1. No	7-2. Yes	7-3. Yes	7-4. Yes	7-5. Yes	8. B
9. B	10. C	11. C	12. B	13. C	14. B	15. A	16. A		

Section C:

1. B	2. A	3. D

Section D:

1-1. Yes	1-2. No	1-3. No	2. B	3. B	4-1. No	4-2. Yes	5-1. No	5-2. Yes	5-3. Yes
6-1. Yes	6-2. Yes	6-3. Yes	6-4. Yes	7. B	8. A	9-1. Yes	9-2. No	9-3. Yes	9-4. No

The responses were calculated as 1 for correct answers and 0 for incorrect or unknown answers.
A total score of four subsections determined the overall nutrition knowledge score.

营养知识问卷计分方式

第一部分:

1-1. 多吃	1-2.少吃	2. A	3-1. 不是	3-2. 不是	3-3. 不是	4. B

第二部分:

1-1. 高	1-2. 高	1-3. 高	2-1. 低	2-2. 高	2-3. 高	2-4. 低	3-1. 高	3-2. 低	4-1. 有
4-2. 没有	4-3. 有	4-4. 有	4-5. 没有	4-6. 有	5-1. 高	5-2. 低	5-3. 低	5-4. 低	5-5. 低
6-1. 低	6-2. 高	6-3. 高	6-4. 高	7-1. 不是	7-2. 是	7-3. 是	7-4. 是	7-5. 是	8. B
9. B	10. C	11. C	12. B	13. C	14. B	15. A	16. A		

第三部分:

1. B	2. A	3. D

第四部分:

1-1. 有关	1-2. 无关	1-3. 无关	2. B	3. B	4-1. 无关	4-2. 有关	5-1. 不是	5-2. 是	5-3. 是
6-1. 是	6-2. 是	6-3. 是	6-4. 是	7. B	8. A	9-1. 是	9-2. 不是	9-3. 是	9-4. 不是

每个小题回答正确计 1 分，回答错误或不确定计 0 分。
每个部分的题目计分相加得到分量表得分，所有题目计分相加得到营养知识总分。
